# Brain Effects Stick Around: PFCs and Neural Cell Development

**Published:** 2008-06

**Authors:** Scott Fields

Although some perfluorinated chemicals—manmade compounds used in such goods as nonstick cookware and stain protectors—are no longer manufactured, decoding the ways in which these chemicals affect humans and wildlife is important because of the stability of the compounds, their persistence in the environment, and their long retention times in living organisms. To date, the biological mechanisms through which these chemicals cause damage have not been well understood. Now, however, researchers have shown that four common perfluorinated chemicals—perfluorooctane sulfonamide (PFOSA), perfluorooctane sulfonic acid (PFOS), perfluorooctanoic acid (PFOA), and perfluorobutane sulfonic acid (PFBS)—affect developing PC12 cells in ways that suggest a variety of mechanisms of action at work **[*EHP* 116:716–722; Slotkin et al.]**.

The team studied whether the chemicals affected the cells’ ability to synthesize DNA, the growth of the cells and their ability to reproduce, cell viability, and the tendency of the cells to manufacture dopamine and acetylcholine neurotransmitters as they differentiated into neural cells. The investigators also assessed the abilities of the chemicals to induce oxidative stress that can damage or kill cells.

For a benchmark comparison, the research team compared the effects of PFOSA, PFOS, PFOA, and PFBS to those of chlorpyrifos, an insecticide and known developmental neurotoxicant. The PC12 cells, a model of neuronal development, were exposed to the chemicals in five concentrations ranging from 10 μM to 250 μM. The effects were assessed over six days—long enough for the cells to differentiate into neuronlike structures.

The researchers demonstrated that all four perfluorinated chemicals affected the PC12 cells. PFOSA had the strongest effects on the health of the cells. Next most significant was PFOS, with PFBS and PFOA tied for third place. Despite similarities in their chemical structures, each chemical had a different effect on neurodevelopment, which suggests the adverse effects are likely mediated by different mechanisms.

PFOSA behaved very differently from PFOA and PFOS. At all concentrations, PFOSA suppressed the production of DNA. At the highest concentration, almost all DNA production was prevented. In addition to PFOSA preventing the production of new cells, existing cells appeared to have been destroyed. Even at the lowest concentration, PFOSA caused more oxidative stress than that seen with a fivefold higher concentration of chlorpyrifos. At the highest PFOSA concentration, cell viability plummeted.

The researchers offer one possible explanation for PFOSA being more toxic than the other perfluorinated chemicals—it is more hydrophobic and therefore crosses cell membranes more easily. This ability to enter cells could indicate that PFOSA and perfluorinated chemicals with similar properties are better able to cross the barriers that guard the placenta and developing brain tissues. Hence, these chemicals may warrant particular research attention.

